# Genotype–Phenotype Correlation in Children: The Impact of *FBN1* Variants on Pediatric Marfan Care

**DOI:** 10.3390/genes11070799

**Published:** 2020-07-15

**Authors:** Veronika C. Stark, Flemming Hensen, Kerstin Kutsche, Fanny Kortüm, Jakob Olfe, Peter Wiegand, Yskert von Kodolitsch, Rainer Kozlik-Feldmann, Götz C. Müller, Thomas S. Mir

**Affiliations:** 1 Pediatric Cardiology, University Heart and Vascular Center Hamburg, Martinistrasse 52, 20246 Hamburg, Germany; flemminghensen@gmx.de (F.H.); j.olfe@uke.de (J.O.); pw95@hotmail.de (P.W.); r.kozlik-feldmann@uke.de (R.K.-F.); go.mueller@uke.de (G.C.M.); mir@uke.de (T.S.M.); 2 Institute of Human Genetics, University Medical Center Hamburg-Eppendorf, Hamburg, Martinistrasse 52, 20246 Hamburg, Germany; kkutsche@uke.de (K.K.); fkortuem@uke.de (F.K.); 3 Cardiology, University Heart and Vascular Center Hamburg, Martinistrasse 52, 20246 Hamburg, Germany; kodolitsch@uke.de

**Keywords:** Marfan syndrome, childhood, genetic testing, *FBN1* variant, genotype–phenotype, variant spectrum

## Abstract

Currently, no reliable genotype–phenotype correlation is available for pediatric Marfan patients in everyday clinical practice. We investigated correlations of *FBN1* variants with the prevalence and age of onset of Marfan manifestations in childhood and differentiated three groups: missense/in-frame, splice, and nonsense/frameshift variants. In addition, we differentiated missense variants destroying or generating a cysteine (cys-missense) and alterations not affecting cysteine. We categorized 105 *FBN1*-positive pediatric patients. Patients with cys-missense more frequently developed aortic dilatation (*p* = 0.03) requiring medication (*p* = 0.003), tricuspid valve prolapse (*p* = 0.03), and earlier onset of myopia (*p* = 0.02) than those with other missense variants. Missense variants correlated with a higher prevalence of ectopia lentis (*p* = 0.002) and earlier onset of pulmonary artery dilatation (*p* = 0.03) than nonsense/frameshift, and dural ectasia was more common in the latter (*p* = 0.005). Pectus excavatum (*p* = 0.007) appeared more often in patients with splice compared with missense/in-frame variants, while hernia (*p* = 0.04) appeared earlier in the latter. Findings on genotype–phenotype correlations in Marfan-affected children can improve interdisciplinary therapy. In patients with cys-missense variants, early medical treatment of aortic dilatation seems reasonable and early regular ophthalmologic follow-up essential. Patients with nonsense/frameshift and splice variants require early involvement of orthopedic specialists to support the growing child.

## 1. Introduction

Marfan syndrome (MFS) is a genetic disorder with a wide clinical phenotype and a reported prevalence of 6.5/100,000 [1]. It is inherited in an autosomal dominant manner. There are some rare cases with compound heterozygous and homozygous *FBN1* variants. Whether patients with biallelic *FBN1* variants are more disease affected than heterozygous cases remains unclear and has been regarded controversial [2]. Decades ago, MFS was diagnosed using the combination of manifestations presenting in different organ systems [3]. Since Dietz et al. revealed the correlation of *FBN1* mutation with this disease, the “modern” diagnosis of MFS based on the Ghent criteria and the later revised Ghent criteria (RGC) became possible [4,5,6]. At present, the RGC enables high-quality, reliable diagnosis of the MFS taking into account the differential diagnoses of other Marfan-like disorders. In addition to the genetic details, it includes a comprehensive clinical catalog of Marfan manifestations that enables diagnosis of the MFS already in the young. Associated with these criteria, the care of affected patients has improved enormously, and survival with almost normal life expectancy has improved [7,8].

With the identification of *FBN1* as the genetic basis of MFS, the hope for a monogenic disease with sufficient prediction of outcome was feasible. However, this has proved difficult, and a prediction of morbidity and mortality of MFS in the individual patient is still lacking [9]. To date, more than 3000 different *FBN1* variants causing MFS have been identified (http://www.umd.be/fbn1/). However, only a few findings about specific gene variants that are correlated with phenotypic aspects have been reported. Variants in the region of exons 24–32 are correlated with a severe form of MFS, called neonatal MFS [10,11]. Detailed information about the individual progress of the disease, with information about the involved organ systems and time of onset of symptoms depending on the specific *FBN1* variant in other Marfan patients, is still lacking [12,13]. However, even though the Marfan genotype may not determine the whole progress of disease alone, information of the genotype effect on the phenotype outcome could improve individualized patient care with respect to the timing of follow-up visits, restriction of physical activity, medical treatment, and elective surgery, as a milestone for Marfan care.

Especially in pediatric Marfan patients, determination of the prognosis and early effective prophylaxis and therapy are essential. Children who have one parent with MFS are often asymptomatic or only mildly affected at the time of genetic diagnosis. The development of typical Marfan features is age-dependent and varies markedly within families [10]. If some genotype–phenotype correlations can be established, then the identification of not only patients at high risk may be possible. Already in the youngest patients, medical prophylaxis could be initiated early to hopefully prevent cardiovascular pathologies causing, to date, the highest morbidity and mortality in MFS. In addition, supporting therapies such as physiotherapy, corset use, or growth management could be applied to significantly improve the quality of life within affected families.

Even though the are many studies analyzing genotype–phenotype correlations in adult Marfan patients, the data in early childhood are lacking. Owing to this, we decided to determine the spectrum of *FBN1* pathogenic variants in our cohort of pediatric Marfan patients to possibly identify relevant clinical aspects in young patients. Especially for “growing children”, individualized patient care would improve the disease outcome. Finally, we review the current knowledge about the genotype–phenotype correlation in pediatric Marfan patients.

## 2. Materials and Methods

### 2.1. Patient Cohort and Data Collection

In this study, we included pediatric patients who had been clinically diagnosed with MFS according to the revised Ghent criteria and carry a heterozygous pathogenic *FBN1* variant (see below). Genetic studies were performed clinically, and informed consent for study and genetic analysis was obtained from the parents or legal guardians of all patients. They gave their informed consent to anonymized use of the genetic findings for scientific purposes. The study was registered, designed, performed, and controlled according to current guidelines of Good Clinical Practice according to the Declaration of Helsinki. Protocol was approved by the local ethics committee (Hamburg, project identification code: PV 4005). We examined all patients in our specialized pediatric Marfan clinic from January 2008 to March 2020.

### 2.2. Genetic Analysis

DNA was isolated from leukocytes by standard procedures. For Sanger sequencing, the coding region and exon/intron boundaries of the *FBN1* gene (NM_000138.4) were amplified from genomic DNA. Amplicons were directly sequenced using the ABI BigDye Terminator Sequencing Kit (Applied Biosystems, Foster City, CA, USA) and an automated capillary sequencer (e.g., ABI 3500; Applied Biosystems). Sequence electropherograms were analyzed using the Sequence Pilot software SeqPatient (JSI Medical Systems, Ettenheim, Germany).

In 2016, Sanger sequencing was replaced by targeted next-generation sequencing (NGS). Enrichment of the regions of interest (ROI) was performed with an Illumina Rapid Capture Custom Enrichment kit or the Illumina Nextera Flex for Enrichment kit, in accordance with the manufacturer’s instructions. Briefly, following the fragmentation of genomic DNA, fragmented DNA was amplified, and patient-specific (index) adapters were added by PCR. Samples from 12 patients were combined into one single hybridization mix containing target-specific capture probes. The DNA-probe hybrids were then captured with streptavidin beads, and nontargeted DNA fragments as well as unspecific binding were removed by heated washes. Next, the captured DNA library was eluted from the beads, purified, and amplified by PCR. The concentration of each library was measured by Qubit fluorometric quantification (Life Technologies, Carlsbad, CA, USA). For the generation of clusters and subsequent sequencing of the targeted DNA samples on a flow cell, a sequencing reagent kit from Illumina was used. High-throughput NGS data were generated on an Illumina sequencing platform. ROI sequences were aligned to the human reference genome (hg19) and visualized and evaluated by using the Sequence Pilot module SeqNext (JSI Medical Systems) [14,15].

We classified *FBN1* sequence variants (according to mRNA reference number NM_000138) as pathogenic or likely pathogenic according to the American College of Medical Genetics and Genomics and the Association for Molecular Pathology standards and guidelines [16]. We did not include variants of unknown significance in this study. We did not subanalyze patients with *FBN1* pathogenic variants located in exons 24–32, as the group was too small for this. To determine the size of the heterozygous deletion encompassing the entire *FBN1* gene in one patient, molecular karyotyping (array comparative genomic hybridization) was carried out using the 180k Agilent array with a mean genome-wide resolution of 100 kb (AMADID#027676, hg19/GRCh37).

### 2.3. Variant Classification

*FBN1* variants were classified into three groups: (1) variants affecting a single or several codons, such as missense variants and in-frame deletions, that leave the reading frame intact; (2) splice site variants, including intronic variants affecting one of the two invariable nucleotides of the splice acceptor or donor site, other intronic variants for which the in silico tools varSEAK (https://varseak.bio/index.php) (JSI Medical Systems) and Human Splicing Finder ver 3.1 (http://umd.be/HSF3/HSF.shtml) [17] predicted an effect on pre-mRNA splicing, and a synonymous variant for which aberrant pre-mRNA splicing has been experimentally demonstrated; and (3) variants introducing a premature stop codon, such as nonsense and frameshift variants, and one deletion of the entire *FBN1* gene, all likely leading to loss of function. Missense variants have been subdivided into those substituting or producing a cysteine and those affecting another highly conserved amino acid residue. We compared the variant groups concerning phenotype presentation and age of manifestation.

### 2.4. Clinical Examination

We examined the clinical manifestations in all patients according to the Ghent Criteria from 1996 and the revised Ghent Criteria (RGC) from 2010 [4,6]. We performed MRI in children as soon as feasible without anesthesia.

An experienced doctor performed echocardiography. We measured the diameter of the sinus of Valsalva (SV) in the parasternal long axis using the leading-edge-to-leading-edge technique at end-diastole. Mitral and tricuspid valves were examined in a four-chamber view, parasternal long axis, and parasternal short axis. Tricuspid valve prolapse (TVP) is defined as valvular thickening and prolapse with chordal elongation involving all three leaflets [18]. Mitral valve prolapse (MVP) in adolescents and adults is defined with leaflet thickening exceeding 5 mm and systolic prolapse of a leaflet into the atrium of more than 2 mm [19]. For younger children, there are no exact values for the definition of MVP. Echocardiography was performed with a Philips diagnostic ultrasound system, EPIQ7 (Amsterdam, The Netherlands), with 12.8 and 5 MHz probes.

### 2.5. Statistical Analysis

We show quantitative data as the mean ± standard deviation. Quantitative variables were investigated with one-way ANOVA followed by Tukey’s multiple comparison test; if the *p*-value was <0.2, additionally an unpaired *t*-test (when comparing three groups) or an unpaired *t*-test or the Welch *t*-test, if necessary (when comparing two groups), was performed. Qualitative variables are given as percentages. Qualitative variables were compared using Fisher’s exact test. A *p*-value of ≤0.05 was assumed to be significant. We collected data with Filemaker software V.10 Pro Advanced (Santa Clara, CA, USA). We performed statistical analyses with Microsoft Excel 2007 (Microsoft, Redmond, WA, USA), SPSS V.23.0 (SPSS, Chicago, IL, USA), and GraphPad Prism 8.4.2 (GraphPad Software Inc, San Diego, CA, USA).

## 3. Results

### 3.1. Clinical Characteristics of Children with MFS

**Figure 1:**
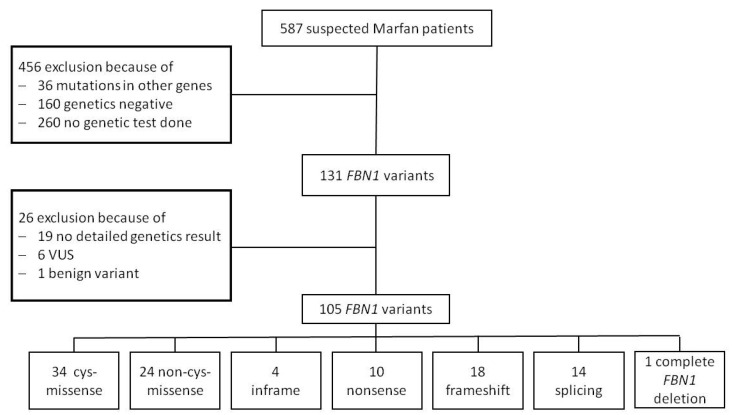
Flow chart of the patient cohort. We examined 587 children and diagnosed MFS in 202 of them. We performed genetic analysis in 327 patients, among whom 131 were *FBN1*-positive. A total of 105 patients with a detailed genetic background were included in our analysis.

We examined 587 children with a mean age of 7.0 ± 5.4 years at the first consultation in our specialized pediatric Marfan clinic, at the Department of Pediatric Cardiology, University Heart and Vascular Center, Hamburg, Germany. We performed genetic analysis in 327 patients and identified *FBN1* variants in 131 of them; 202 of the 327 children had a clinical diagnosis of MFS according to RGC. Thus, detection rate of FBN1 in Marfan children with clinically diagnosed MFS according RGC was 64.9%. The mean age at genetic diagnosis was 7.8 ± 5.4 years. For detailed variant analysis, we included 105 children (55 males, 52.4%). We excluded six patients with variants of uncertain significance and one with a benign *FBN1* variant. In 16 patients, genetic analysis was performed externally, and we did not have the detailed findings. Nine patients showed FBN1 variant for neonatal MFS. Thereby, 11.8% of cys-missense, 8.3% of non-cys-missense, 11.1% of frameshift, and 10% of nonsense variants were neonatal variants. We did not analyze those patients in detail as the numbers were too low for statistical analysis. A flow chart of our patient cohort is shown in Figure 1.

**Figure 2:**
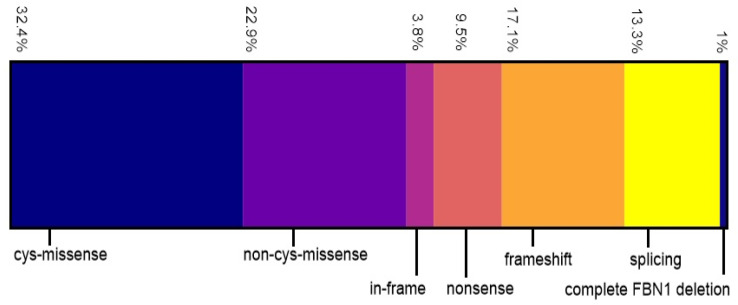
Type and percentage of *FBN1* variants identified in our cohort (*n* = 105): missense affecting a cysteine residue (cys-missense; blue), missense not affecting a cysteine residue (non-cys-missense; purple), in-frame (pink), nonsense (light red), frameshift (orange), splicing (yellow), entire *FBN1* gene deletion (dark blue).

Patient data on the prevalence and age of onset of clinical Marfan findings are summarized in Table 1. Of 105 probands, 70 (66.7%) had family histories of MFS, 23 (21.9%) were sporadic, and the other 12 (11.4%) patient family histories were not available. The distribution of different variant types is shown in Figure 2. Missense variants were the most common variant type, found in 58 patients (55.3%), among which 34 (58.6%) involved a cysteine residue (cys-missense). With decreasing frequency, we also identified frameshift (17.1%), splicing (13.3%), nonsense (9.5%), and in-frame (3.8%) variants as well as deletion of the whole *FBN1* gene (1%).

Concerning the main criteria for MFS, patients with cysteine-affecting missense variants showed the highest prevalence of aortic dilatation (67.6%) and ectopia lentis (EL) (44.1%). Patients with nonsense/frameshift variants showed the highest rate (44.8%) of a positive systemic score.

### 3.2. Genotype–Phenotype Correlations

**Figure 3:**
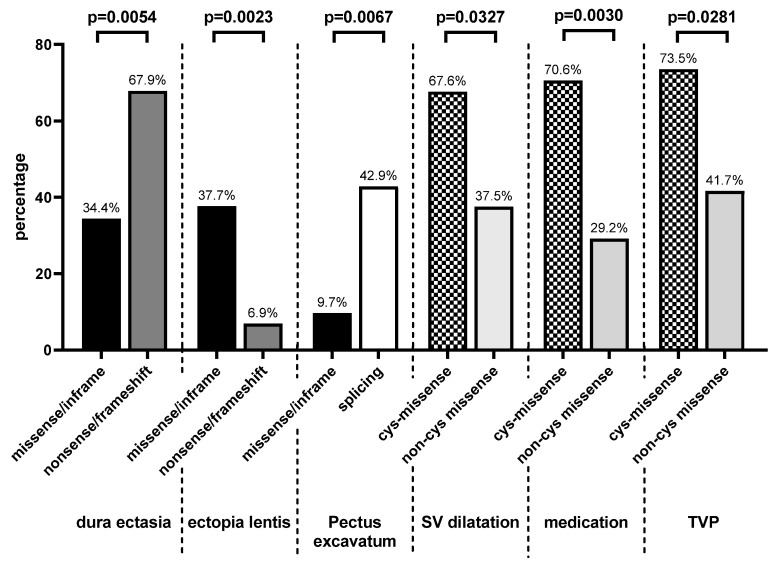
Dural ectasia is more common in patients with nonsense/frameshift than missense/in-frame. Ectopia lentis is more common in patients with missense/in-frame than nonsense/frameshift. Pectus excavatum is more common in patients with splicing than missense/in-frame. More cardiovascular (SV dilatation, dilatation of sinus Valsalva; medication; TVP, tricuspid valve prolapse) in patients with missense variants involving cysteine (cys-missense) than missense variants without cysteine involvement (non-cys missense).

**Figure 4:**
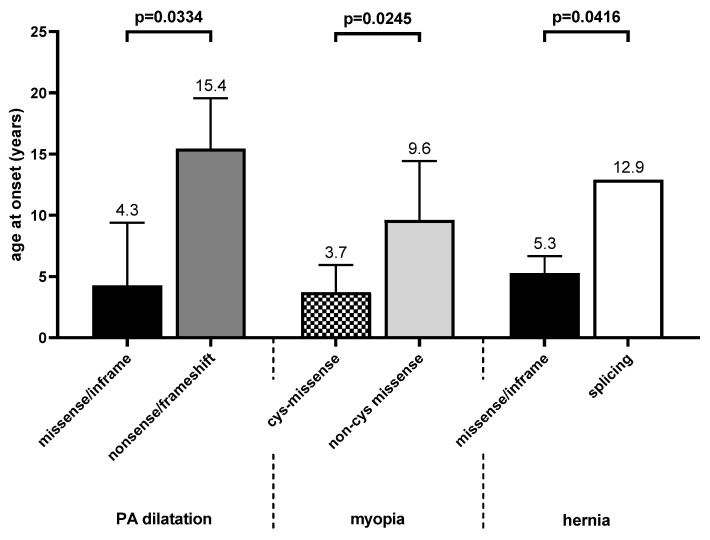
Pulmonary artery (PA) dilatation appears earlier in missense than in nonsense/frameshift variants. Myopia appears earlier in patients with cys-missense than non-cys missense. Hernia appears earlier in patients with missense/in-frame than splicing.

We analyzed the genotype–phenotype correlation for different *FBN1* variants. Significant data are summarized in Table 2, and the findings are visualized in Figure 3 and Figure 4. In all compared groups, positive family history did not differ significantly. Detailed data for all Marfan features are shown in the Supplemental Material.

When comparing missense/in-frame variants with splicing variants (Table S1), pectus excavatum (*p* = 0.0067) was more common in pediatric patients with splicing variants. In addition, hernia appeared earlier in patients with missense/in-frame variants than in those with splicing variants (*p* = 0.04).

In the comparison of the missense/in-frame variants with the nonsense/frameshift *FBN1* variants (Table S2), age of onset of pulmonary artery dilatation was earlier in patients with missense/in-frame *FBN1* variants (*p* = 0.0334). Dural ectasia (*p* = 0.0054) was more common in patients with nonsense/frameshift variants than in patients with missense/in-frame variants. In contrast, the prevalence of EL was higher in patients with missense/in-frame variants than in those with nonsense/frameshift *FBN1* variants (*p* = 0.002).

We did not find significant differences between nonsense/frameshift and splicing variants in any of the analyzed clinical Marfan features concerning prevalence or age of onset (Table S3).

Patients with *FBN1* variants affecting a cysteine residue showed a higher rate of SV dilatation (*p* = 0.033) and tricuspid valve prolapse (*p* = 0.03) than patients with variants not involving a cysteine residue. In addition, medication was initiated more often in cys-missense group (*p* = 0.003). EL appeared to be more common and with an earlier onset in patients with variants involving a cysteine; however, this was not significant. Myopia appeared significantly earlier in Marfan patients with cysteine-affected variants (*p* = 0.025). Moreover, pneumothorax appeared earlier (*p* = 0.02) in patients harboring variants without cysteine involvement, but the number of affected patients in both groups was too low. Skeletal features and dural ectasia did not show relevant differences between patients with variants with and without cysteine involvement. Data on the comparison of patients with missense variants with and without cysteine involvement are shown in Table S4.

## 4. Discussion

Many studies have analyzed the genotype–phenotype correlation in MFS, but detailed information on this in pediatric patients has rarely been published. There is a lack of information on the correlations between variant types and the individual organ manifestations in childhood. In our study, we concentrated on the clinical presentation of pediatric Marfan patients and its correlation to *FBN1* variants. Our *FBN1* detection rate in children being diagnosed with MFS according the RGC was 64.9%. This matches with previously reported data where FBN1 detection rate of RGC-positive patients was almost similar, while some studies described a slightly higher detection rate [20,21,22]. Since we also diagnosed other genes from Marfan-like diseases in addition to FBN1 variants, the actual detection rate of a causative gene mutation of a superordinate connective tissue disease is higher. If we summarize all genes for Marfan-like diseases, the detection rate would be 82.7%.

In our pediatric group, we mainly identified missense *FBN1* variants, with the majority affecting a cysteine residue. Robinson et al. summarized the first genotype–phenotype correlations in a large MFS cohort almost 20 years ago and differentiated the variant types. They also found that missense variants were the most common [23]. Other studies confirmed this distribution of variants. Missense variants substituting or creating a cysteine in one of the cbEGF domains (calcium-binding EGF) are the most common [24,25,26,27]. Overall, the distribution of pathogenic variants identified in our cohort is consistent with previous data [28,29,30].

To put the classification of respective variants into context regarding the previously published studies, we would like to review the possible variant classification system. In the last 20 years, many researchers differentiated pathogenic FBN1 variants causing a “reduced number” (quantitative) and those causing a “defective population” (qualitative) of microfibrils [31,32]. In this regard, haploinsufficient (HI) variants cause a reduced amount of fibrillin−1 and included nonsense and frameshift variants, which presumably lead to nonsense-mediated mRNA decay [33]. Others have also described those variants as truncating or premature terminating codons (PTCs), in terms of the effect of the variant on the protein level. In contrast, dominant negative (DN) variants (missense variants, in-frame insertion or deletion, in-frame exon skipping variants) result in a structurally modified protein, which competes with the wild-type protein expressed from the normal *FBN1* allele.

We decided to compare the detailed variant types. We classified variants that (1) leave the reading frame intact (missense, in-frame), (2) splice variants, and (3) variants introducing a premature stop codon (nonsense, frameshift variants, deletion of the entire *FBN1* gene), all likely leading to loss of function. Concerning missense variant, we differentiated the cases with and without cysteine involvement. In the following, we bring our results into context with the existing literature. Only a few studies have exclusively analyzed children (Table 3). Most of the existing findings on phenotype–genotype correlations are focused on adults or just include some children in the subject group. Only in a few studies was a subanalysis of the pediatric group performed. In Table 3, we summarize studies performed to date that analyzed or performed subgroup analysis of children with MFS and complement our data.

Tables providing overall reviews of the cardiovascular, ocular, skeletal, pulmonary, skin, and dural manifestations of MFS and the described genotype–phenotype correlations are presented in the Supplemental Material (Tables S5–S7).

### 4.1. Cardiovascular Genotype–Phenotype Correlations

Patients with cysteine-affecting missense variants showed a higher prevalence of SV dilatation and tricuspid valve prolapse than those without cysteine involvement in our cohort. Accordingly, drug treatment was initiated more often in this group. Detaint et al. also found a higher risk for aortic dilatation or events and mitral valve prolapse in patients with variants altering a cysteine [34]. Moreover, Seo et al. and Gao et al. presented consistent results concerning aortic root dilatation [35,36].

We did not find a significant difference concerning cardiovascular manifestations in patients with missense/frameshift variants compared with those with splicing changes. However, Baudhuin et al. identified a higher rate of splicing variants in patients with aortic involvement [28].

We did not identify any significant aortic genotype–phenotype correlations for nonsense/frameshift variants in comparison to missense/in-frame variants [30,37]. Many other researchers showed higher cardiovascular involvement in the nonsense/frameshift group in adult or mixed study cohorts [27,28,29,36,38]. Franken et al. showed a more severe aortic phenotype and a higher risk for aortic mortality in patients with HI variant, which represents the nonsense/frameshift variants [39]. In addition, in HI patients, treatment with losartan appeared to be more efficient [40]. Moreover, Xu et al. and Li et al. showed a higher rate of aortic dissection in this patient group [22,26]. The causes of these results could be obscured in childhood, as aortic dissection is rare in the young. We showed an earlier onset of pulmonary artery dilatation in patients with missense variants compared with that in those with nonsense/frameshift variants. This has not been investigated previously.

In summary, there appears to be a consistent trend of a higher probability of aortic disease in patients with variants involving cysteine. Although we did not identify this in our young cohort, there is a higher risk for aortic involvement in patients with nonsense/frameshift variants (Table S5).

### 4.2. Ocular Genotype–Phenotype Correlations

With respect to ocular manifestations, we found significantly more patients with EL in the missense/in-frame group than among those with nonsense/frameshift variants. This is consistent with other studies [9,35]. We also found a trend for more and earlier manifestations of EL in patients with missense/in-frame variants in comparison with the findings in those with splicing variants, although this was not significant. Myopia also appeared earlier in patients with cysteine-affecting than in those with non-cysteine-affecting missense variants. In terms of the prevalence and onset of EL in our missense-cysteine-affected patients, EL was more common and appeared earlier than in the non-cysteine-affected group, in which this was not significant. Schrijver et al. reported more EL in patients with missense variants involving a cysteine residue [41]. Many other groups also revealed findings consistent with this [20,23,30,42,43].

In summary, the data about EL are concordant. EL is more common in Marfan patients with missense variants and especially in those with cysteine-involving missense variants (Table S6).

### 4.3. Skeleton, Dura, Lung, and Skin Genotype–Phenotype Correlations

In our cohort, patients with nonsense/frameshift variants showed the highest rate of a positive systemic score. Overall, we saw a trend for higher skeletal involvement in patients with nonsense/frameshift variants than in those with missense/in-frame variants, but this did not reach significance. This is consistent with previous data which also showed significant higher skeletal involvement in patients with nonsense/frameshift variants, respectively premature termination codons or HI variants, which are caused by nonsense and frameshift variants. Haine et al. showed more severe musculoskeletal involvement (i.e., arachnodactyly, scoliosis, pectus deformity, joint laxity) in those with *FBN1* premature termination codon variant than in patients with in-frame variant [44]. Faivre et al. also showed higher involvement of skeleton and skin in patients with PTC than in those with in-frame variants [43]. In addition, Comeglio et al. and Schrivjer et al. showed higher skeletal involvement in patients with PTC variants [45,46]. Moreover, Franken et al. identified a higher prevalence of pectus carinatum in HI patients, which also included nonsense/frameshift variants [40]. Age-dependent onset of Marfan features may explain missing significance of our data. In addition, we found significantly more pectus excavatum in patients with splice variants than in those with missense/in-frame variants. Such skeletal findings with age dependence could help in establishing appropriate prophylactic treatment and providing appropriate support for Marfan-affected children at various ages. This could be an approach concerning prophylactic treatment and support of the growing child.

We also identified hernias earlier in the missense/in-frame group than in the splicing group. In our cohort, dural ectasia was more prevalent in patients with nonsense/frameshift variants than in those with missense/in-frame variants. This was also identified in previous studies [35,40].

Pneumothorax appeared earlier in the missense group without cysteine involvement, whereas our cohort of pneumothorax patients is too small to determine an accurate genotype–phenotype correlation (total of five patients). Other data about pneumothorax–genotype correlation are missing.

In summary, there are consistent findings on a severe skeletal phenotype in patients with nonsense/frameshift variants (representing HI and PTC), whereas data in childhood are rare [44]. Dural ectasia also appears to be more common in patients with nonsense/frameshift variants (Table S7).

In summary, we again showed that similar *FBN1* variants can cause a broad variety of Marfan phenotypes from very mild forms to fatal pathologies, with known inter- and intrafamilial variability [48,49]. *FBN1* variants are often unique, so nearly every family has its own variant [23]. However, we identified some reliable findings on the genotype–phenotype correlation consistent with the literature. After many studies have been carried out on the genotype–phenotype correlations, it seems likely that the genotype alone does not determine the disease of the MFS and its course. Nevertheless, we should use the obtained information about genotype–phenotype correlation in everyday clinical practice. Especially in childhood, prophylactic treatment can be useful for the pathologies of MFS. This does not only apply to the early identification and treatment of cardiovascular manifestations. Skeletal pathologies can benefit from supportive therapy, providing a better outcome with a better quality of life.

In the context of MFS, accurate determination of the genotype–phenotype correlation would be a major milestone. However, the influence of the pathogenic *FBN1* variant on the resulting phenotype seems to be bigger, and the underlying *FBN1* variant seems not to determine the progress of disease alone [33]. Findings have extended our understanding of the genotype–phenotype correlation in the last few years. First, it was recently determined that the assumedly isolated architectural dysfunction of fibrillin−1 was actually enhanced by a biological dysfunction with a dysregulation of transforming growth factor-β (TGFβ) [50,51]. The effect of the TGFβ metabolism in combination with different *FBN1* variants has been insufficiently investigated and could be another piece of the puzzle in clarifying the genotype–phenotype correlation of the disease. Measuring TGFβ levels could be useful in this regard. Second, other genetic modifiers and environmental factors seem to play an important role in the course of disease. Genome-wide analyses of other genetic aspects that may modify the *FBN1* gene have been started in adults [49]. Benarroch et al., for example, identified sarcolipin and calcium as potential transregulators for *FBN1* [52]. Third, it is possible that the range of materials to be analyzed for judging the course and outcome of the disease needs to be extended. For example, studies have analyzed skin fibroblasts in adults to identify different phenotypes [49,52,53,54]. Finally, we have not yet developed the perfect care package for Marfan patients, but the care for Marfan syndrome has already substantially improved in the last 30 years. The challenge for the next 30 years may be to develop individualized diagnosis and therapy.

### 4.4. Study Limitations

The present study has several limitations. First, this is a retrospective study with some lack of information. Genetic analysis was not always performed in our department, and detailed information was sometimes missing. Thus, we had to exclude 19 patients from our study. This significantly reduces the number of children in our group. Second, we included nine children with neonatal MFS in our collective who are known to be more affected of MFS. Thereby, the distribution of neonatal MFS in the cys-missense, non-cys-missense, frameshift, and nonsense variant group was almost equal, so the impact of the statistical result should be small. Third, we assumed onset of pathologies of MFS as the time of its clinical diagnosis, which is probably not the true onset. As we re-examined the patients in every visit it may be approximately correct. At last, some variant groups are very small and thus have a statistical strength deficiency. Additionally, some Marfan manifestations, especially aspects of the systemic manifestation, are rare in children, and it is thus hard to differentiate significant results between variant groups.

## 5. Conclusions

The genotype appears to play a crucial, if not the only, role in MFS outcome. Even though the genotype–phenotype correlation for MFS is still lacking, we can use some consistent findings on this condition in everyday clinical practice. For example, according to our data, patients with cysteine-involving missense variants, and according to previous studies on nonsense/frameshift variants, require early medical treatment for aortic or cardiac involvement. Those with missense variants, particularly those involving cysteine, require sufficient ophthalmologic support. In addition, in patients with nonsense/frameshift variants, there is a need for early orthopedic involvement to potentially prevent some skeletal malformations in the growing child.

Knowledge of the genotype–phenotype correlation can promote individualized care in MFS. The findings that we describe can particularly improve care for children with MFS, in whom Marfan features develop over time and for whom some intervention is possible. Such findings can also facilitate decision-making about who to involve in the interdisciplinary Marfan care team in potentially unaffected children with a pathogenic *FBN1* variant.

## Figures and Tables

**Table 1:** Patient data and clinical Marfan findings in pediatric Marfan patients with *FBN1* variants (*n* = 105).

Variables	All patients (*n* = 105)	Age of onset (years)
Age at first consultation (years)	7.0 ± 5.4	
Sex	55 males (52.4%), 50 females (47.6%)	
Age at genetic diagnosis (years)	7.8 ± 5.4	
Medications	64 (61.0%)	8.4 ± 5.0
First-grade family history positivity	70 (66.7%)	
**Cardiovascular features**		
Sinus of Valsalva dilatation	60 (57.1%)	7.9 ± 5.6
Mitral valve prolapses	58 (55.2%)	8.6 ± 5.3
Tricuspid valve prolapses	70 (67.3%)	7.6 ± 5.4
Pulmonary artery dilatation	10 (9.6%)	7.8 ± 7.0
Surgical aortic root replacement	6 (5.7%)	11.7 ± 6.7
**Skeletal features**		
Dural ectasia	47 (46.5%)	11.7 ± 4.4
High arched palate	61 (58.1%)	9.3 ± 5.0
Facial dysmorphism	33 (31.4%)	6.1 ± 5.0
Arm-span-to-height ratio > 1.05	33 (31.4%)	11.0 ± 4.5
Pectus excavatum	19 (18.1%)	8.5 ± 4.9
Pectus carinatum	24 (22.9%)	10.6 ± 4.4
Scoliosis	38 (36.2%)	10.0 ± 4.8
Wrist and thumb sign	17 (16.2%)	10.9 ± 4.4
Foot deformity	59 (56.2%)	7.9 ± 4.9
Reduced elbow extension	12 (11.4%)	10.9 ± 6.1
**Ocular features**		
Ectopia lentis	29 (27.6%)	7.9 ± 5.5
Myopia	22 (21.2%)	6.3 ± 4.5
Other features		
Skin striae	24 (22.9%)	13.5 ± 3.1
Hernia	8 (7.6%)	7.2 ± 3.5
Pneumothorax	5 (4.8%)	15.0 ± 0.6
Systemic score ≥ 7	38 (36.2%)	11.1 ± 5.0

**Table 2:** Significant genotype–phenotype findings in patients with MFS (PA dilatation, dilatation of pulmonary artery; SV dilatation, dilatation of sinus of Valsalva; TVP, tricuspid valve prolapse).

	Prevalence			Age of Onset		
	Missense/in-Frame	Splicing	*p*-Value	Missense/in-Frame	Splicing	*p*-Value
Pectus excavatum	6/62 (9.7%)	6/14 (42.9%)	0.0067			
Hernia				5.3 ± 1.4	12.9 ± 0.0	0.0416
	**Missense/in-frame**	**Nonsense/frameshift**	***p*-value**	**Missense/in-frame**	**Nonsense/frameshift**	***p*-value**
PA dilatation				4.3 ± 5.1	15.4 ± 4.1	0.0334
Dural ectasia	21/61 (34.4%)	19/28 (67.8%)	0.0054			
Ectopia lentis	23/62 (37.1%)	2/29 (6.9%)	0.0023			
	**Cys-missense**	**Missense cysteine not involved**	***p*-value**	**Cys-missense**	**Missense cysteine not involved**	***p*-value**
SV dilatation	23/34 (67.6%)	9/24 (37.5%)	0.0327			
TVP	25/34 (73.5%)	10/24 (41.7%)	0.0281			
Medication	24/34 (70.6%)	7/24 (29.2%)	0.003			
Myopia				3.7± 2.2	9.6 ± 4.8	0.0245

**Table 3:** Genotype–phenotype correlation in children. (YOP, year of publication; SV-Dil, Dilatation of sinus of Valsalva; PA-Dil, pulmonary artery dilatation; EL, ectopia lentis; cys-missense, missense variants involving a cysteine; non-cys-missense, missense variants not involving a cysteine).

Authors	YOP	Results
Loeys et al.	2001	Subanalysis, 38 children, no significant findings concerning phenotype (especially no findings on cysteine involvement and EL, cardiovasc.)
Arbustini et al.	2005	Subanalysis, 30 children, more EL in cys-missense vs. non-cys-missense, some trends, not significant
Faivre et al.	2009	302 children, comparison of neonatal MFS vs. other variants
Faivre et al.	2009	FBN1 variant analysis in neonatal MFS
Stheneur et al. [47]	2011	Neonatal MFS
Pees et al.	2014	49 children, exons 1–21: 80% ectopia lentis exons 23–32: higher probability of aortic root dilatation
Haine et al.	2015	48 patients (5.3–25.2 years). More musculoskeletal involvement in PTC than in-frame
Seo et al.	2018	Subanalysis, 12 children, no clinical difference in cys-missense vs. non-cys-missense
Stark et al.	2020	105 children More SV-Dil, TVP, medication in cys-missense than in non-cys-missense Earlier PA-Dil in missense/in-frame than in nonsense/frameshift More EL in missense than in nonsense/frameshift Earlier myopia in cys-missense than in non-cys-missense More pectus excavatum in splicing than in missense/in-frame Earlier hernias in missense/in-frame than in splicing More dural ectasia in nonsense/frameshift than in missense

## Supplementary Materials

The following are available online at https://www.mdpi.com/2073-4425/11/7/799/s1, Table S1: Missense/in-frame vs. splicing mutation, Table S2: Missense/in-frame vs. nonsense/frameshift mutations, Table S3: Nonsense/frameshift mutations vs. Splicing mutations, Table S4: Missense mutations involving a cysteine (cys-missense) vs. Missense mutations not involving a cysteine (non-cys-missense), Table S5: Cardiovascular genotype-phenotype correlations, Table S6: Ocular genotype-phenotype correlations, Table S7: Skeleton, skin, lung and dural genotype-phenotype correlations.
