# Supplementary material for: Genotype–Phenotype Correlation in Children: The Impact of FBN1 Variants on Pediatric Marfan Care
Source: Genes (Basel). 2020 Jul 15;11(7):799. doi: 10.3390/genes11070799 (PMC7397236; doi:10.3390/genes11070799)
Supplement: Supplementary file 1 [file genes-11-00799-s001.zip › genes-855487-supplementary tables.pdf]

**Supplementary Materials**

**Table S1** Missense/ in-frame vs. splicing mutation (SV, sinus of Valsalva; MVP, mitral valve prolapse; TVP, tricuspid valve prolapse, PA dilatation, pulmonary artery dilatation; David, David surgery; Facial features (>3 of enophthalmus, dolichocephaly, downslanting palpebral fissures, malar hypoplasia, retrognathia), foot deformity (pes planus or hindfoot deformity).

| | Prevalence | | | Age of onset | | |
|---|---|---|---|---|---|---|
| | Missense/ in-frame | Splicing | p-value | Missense/ in-frame | Splicing | p-value |
| SV dilatation | 35/62 (56.5%) | 7/14 (50.0%) | ns | 7.4 ± 0.5 | 9.4 ± 6.9 | ns |
| MVP | 31/62 (50.0%) | 9/14 (64.3%) | ns | 7.8 ± 5.7 | 10.0 ± 5.9 | ns |
| TVP | 38/62 (61.3%) | 11/14 (78.6%) | ns | 6.9 ± 5.6 | 10.0 ± 6.1 | ns |
| PA dilatation | 6/61 (9.8%) | 2/14 (14.3%) | ns | 4.3 ± 5.1 | 10.5 ± 9.1 | ns |
| David | 5/62 (8.1%) | 0/14 (0.0%) | ns | 13.1 ± 5.5 | - | - |
| Medication | 35/62 (56.5%) | 8/14 (57.1%) | ns | 7.6 ± 5.1 | 11.5 ± 4.1 | ns |
| Dural ectasia | 21/61 (34.4%) | 7/14 (50.0%) | ns | 11.1 ± 4.9 | 14.7 ± 3.4 | ns |
| High arched palate | 33/62 (53.2%) | 9/14 (64.3%) | ns | 9.0 ± 5.1 | 11.4 ± 4.8 | ns |
| Facial feature | 24/62 (38.7%) | 3/14 (21.4%) | ns | 5.9 ± 5.8 | 5.5 ± 3.3 | ns |
| Arm-span-to-height ratio > 1.05 | 15/62 (24.2%) | 5/14 (35.7%) | ns | 11.0 ± 4.7 | 10.2 ± 5.9 | ns |
| Pectus excavatum | 6/62 (9.7%) | 6/14 (42.9%) | **0.0067** | 7.5 ± 6.7 | 7.7 ± 4.4 | ns |
| Pectus carinatum | 15/62 (24.2%) | 2/14 (14.3%) | ns | 10.4 ± 4.9 | 14.7 ± 2.6 | ns |
| scoliosis | 22/62 (35.5%) | 5/14 (35.7%) | ns | 10.0 ± 4.7 | 10.9 ± 5.2 | ns |
| Wrist and thumb sign | 7/62 (11.3%) | 3/14 (21.4%) | ns | 10.5 ± 4.6 | 9.2 ± 6.7 | ns |
| Foot deformity | 35/62 (56.5%) | 7/14 (50.0%) | ns | 7.6 ± 5.5 | 8.8 ± 4.7 | ns |
| Reduced elbow-extension | 9/62 (14.5%) | 0/14 (0.0%) | ns | 10.5 ± 6.8 | - | ns |
| Ectopia lentis | 23/62 (37.1%) | 4/14 (28.6%) | ns | 7.4 ± 5.5 | 10.1 ± 6.4 | ns |
| Myopia | 14/61 (23.0%) | 3/14 (21.4%) | ns | 6.3 ± 4.4 | 7.5 ± 7.2 | ns |
| Skin striae | 14/62 (22.6%) | 4/14 (28.6%) | ns | 13.4 ± 3.4 | 13.1 ± 4.1 | ns |
| Hernia | 3/62 (4.8%) | 1/14 (7.1%) | ns | 5.3 ± 1.4 | 12.9 ± 0.0 | **0.0416** |
| pneumothorax | 4/62 (6.5%) | 0/14 (0%) | ns | 15.1 ± 0.6 | - | ns |
| Systemic score ≥ 7 | 21/59 (35.6%) | 4/14 (28.6%) | ns | 11.1 ± 5.5 | 14.1 ± 2.1 | ns |

**Table S2** Missense/ in-frame vs. nonsense/ frameshift mutations (SV, sinus of Valsalva; MVP, mitral valve prolapse; TVP, tricuspid valve prolapse, PA dilatation, pulmonary artery dilatation; David, David surgery; Facial features (>3 of enophthalmus, dolichocephaly, downslanting palpebral fissures, malar hypoplasia, retrognathia), foot deformity (pes planus or hindfoot deformity).

| | Prevalence | | | Age of onset | | |
|---|---|---|---|---|---|---|
| | Missense/ in-frame | Nonsense/ frameshift | p-value | Missense/ in-frame | Nonsense/ frameshift | p-value |
| SV dilatation | 35/62 (56.5%) | 18/29 (62.1%) | ns | 7.4 ± 0.5 | 8.5 ± 4.5 | ns |
| MVP | 31/62 (50.0%) | 18/29 (62.1%) | ns | 7.8 ± 5.7 | 9.1 ± 4.0 | ns |

| | | | | | | |
|---|---|---|---|---|---|---|
| TVP | 38/62 (61.3%) | 21/28 (75.0%) | ns | 6.9 ± 5.6 | 7.6 ± 4.6 | ns |
| PA dilatation | 6/61 (9.8%) | 2/29 (6.9%) | ns | 4.3± 5.1 | 15.4 ±4.1 | **0.0334** |
| David | 5/62 (8.1%) | 1/29 (3.4%) | ns | 13.1±5.5 | 13.5 ± 0.0 | ns |
| Medication | 35/62 (56.5%) | 21/29 (72.4%) | ns | 7.6 ± 5.1 | 8.7 ± 4.9 | ns |
| Dural ectasia | 21/61 (34.4%) | 19/28 (67.8%) | **0.0054** | 11.1 ± 4.9 | 11.1 ± 3.8 | ns |
| High arched palate | 33/62 (53.2%) | 19/29 (65.5%) | ns | 9.0 ± 5.1 | 8.9 ± 4.7 | ns |
| Facial feature | 24/62 (38.7%) | 6/29 (20.7%) | ns | 5.9 ± 5.8 | 7.2 ± 2.3 | ns |
| Arm-span-to-height ratio > 1.05 | 15/62 (24.2%) | 13/29 (44.8%) | ns | 11.0 ± 4.7 | 11..4 ± 4.0 | ns |
| Pectus excavatum | 6/62 (9.7%) | 7/29 (24.1%) | ns | 7.5 ± 6.7 | 9.9 ± 4.1 | ns |
| Pectus carinatum | 15/62 (24.2%) | 7/29 (24.1%) | ns | 10.4 ± 4.9 | 9.9 ± 3.4 | ns |
| scoliosis | 22/62 (35.5%) | 11/29 (37.9%) | ns | 10.0 ± 4.7 | 9.7 ± 5.3 | ns |
| Wrist and thumb sign | 7/62 (11.3%) | 7/29 (24.1%) | ns | 10.5 ± 4.6 | 11.9 ± 3.2 | ns |
| Foot deformity | 35/62 (56.5%) | 17/29 (58.6%) | ns | 7.6 ± 5.5 | 8.1 ± 4.0 | ns |
| Reduced elbow-extension | 9/62 (14.5%) | 3/28 (10.7%) | ns | 10.5 ± 6.8 | 11.8 ± 4.8 | ns |
| Ectopia lentis | 23/62 (37.1%) | 2/29 (6.9%) | **0.0023** | 7.4 ± 5.5 | 9.1 ± 4.9 | ns |
| Myopia | 14/61 (23.0%) | 5/29 (17.2%) | ns | 6.3 ± 4.4 | 5.5 ± 4.1 | ns |
| Skin striae | 14/62 (22.6%) | 6/29 (20.7%) | ns | 13.4 ± 3.4 | 14.1 ± 2.1 | ns |
| Hernia | 3/62 (4.8%) | 4/29 (13.8%) | ns | 5.3 ± 1.4 | 7,.3 ± 3.5 | ns |
| pneumothorax | 4/62 (6.5%) | 1/29 (3.4%) | ns | 15.1 ± 0.6 | 14.5 ± 0.0 | ns |
| Systemic score ≥ 7 | 21/59 (35.6%) | 13/29 (44.8%) | ns | 11.1 ± 5.5 | 11.3 ±4.1 | ns |

**Table S3** Nonsense/ frameshift mutations vs. Splicing mutations (SV, sinus of Valsalva; MVP, mitral valve prolapse; TVP, tricuspid valve prolapse, PA dilatation, pulmonary artery dilatation; David, David surgery; Facial features (>3 of enophthalmus, dolichocephaly, downslanting palpebral fissures, malar hypoplasia, retrognathia), foot deformity (pes planus or hindfoot deformity).

| | Prevalence | | | Age of onset (years) | | |
|---|---|---|---|---|---|---|
| | Nonsense/ frameshift | Splicing | p-value | Nonsense / frameshift | Splicing | p-value |
| SV dilatation | 18/29 (62.1%) | 7/14 (50.0%) | ns | 8.5 ± 4.5 | 9.4 ± 6.9 | ns |
| MVP | 18/29 (62.1%) | 9/14 (64.3%) | ns | 9.1 ± 4.0 | 10.0 ± 5.9 | ns |
| TVP | 21/28 (75.0%) | 11/14 (78.6%) | ns | 7.6 ± 4.6 | 10.0 ± 6.1 | ns |
| PA dilatation | 2/29 (6.9%) | 2/14 (14.3%) | ns | 15.4 ± 4.1 | 10.5 ± 9.1 | ns |
| BAV | 1/28 (3.5%) | 0/14 (0%) | ns | 17.7 ± 0.0 | - | - |
| Medication | 21/29 (72.4%) | 8/14 (57.1%) | ns | 8.7 ± 4.9 | 11.5 ± 4.1 | ns |
| Dural ectasia | 19/28 (67.8%) | 7/14 (50.0%) | ns | 11.1 ± 3.8 | 14.7 ± 3.4 | ns |
| High arched palate | 19/29 (65.5%) | 9/14 (64.3%) | ns | 8.9 ± 4.7 | 11.4 ± 4.8 | ns |
| Facial feature | 6/29 (20.7%) | 3/14 (21.4%) | ns | 7.2 ± 2.3 | 5.5 ± 3.3 | ns |
| Arm-span-to-height ratio > 1.05 | 13/29 (44.8%) | 5/14 (35.7%) | ns | 11.4 ± 4.0 | 10.2 ± 5.9 | ns |
| Pectus excavatum | 7/29 (24.1%) | 6/14 (42.9%) | ns | 9.9 ± 4.1 | 7.7 ± 4.4 | ns |

| | | | | | | |
|---|---|---|---|---|---|---|
| Pectus carinatum | 7/29 (24.1%) | 2/14 (14.3%) | ns | 9.9 ± 3.4 | 14.7 ± 2.6 | ns |
| scoliosis | 11/29 (37.9%) | 5/14 (35.7%) | ns | 9.7 ± 5.3 | 10.9 ± 5.2 | ns |
| Wrist and thumb sign | 7/29 (24.1%) | 3/14 (21.4%) | ns | 11.9 ± 3.2 | 9.2 ± 6.7 | ns |
| Foot deformity | 17/29 (58.6%) | 7/14 (50.0%) | ns | 8.1 ± 4.0 | 8.8 ± 4.7 | ns |
| Reduced elbow-extension | 3/28 (10.7%) | 0/14 (0%) | ns | 11.8 ± 4.8 | - | ns |
| Ectopia lentis | 2/29 (6.9%) | 4/14 (28.6%) | ns | 9.1 ± 4.9 | 10.1± 6.4 | ns |
| Myopia | 5/29 (17.2%) | 3/14 (21.4%) | ns | 5.5 ± 4.1 | 7.5 ± 7.2 | ns |
| Skin striae | 6/29 (20.7%) | 4/14 (28.6%) | ns | 14.1 ± 2.1 | 13.1 ± 4.1 | ns |
| Hernia | 4/29 (13.8%) | 1/14 (7.1%) | ns | 7.3 ± 3.5 | 12.9 ± 0.0 | ns |
| pneumothorax | 1/29 (3.4%) | 0/14 (0%) | ns | 14.6 ± 0.0 | - | ns |
| Systemic score ≥ 7 | 13/29 (44.8%) | 4/14 (28.6%) | ns | 11.3 ± 4.1 | 14.1 ± 2.1 | ns |

**Table S4** Missense mutations involving a cysteine (cys-missense) vs. Missense mutations not involving a cysteine (non-cys-missense) (SV, sinus of Valsalva; MVP, mitral valve prolapse; TVP, tricuspid valve prolapse, PA dilatation, pulmonary artery dilatation; David, David surgery; Facial features (>3 of enophthalmus, dolichocephaly, downslanting palpebral fissures, malar hypoplasia, retrognathia), foot deformity (pes planus or hindfoot deformity).

| | Prevalence | | | Age of onset | | |
|---|---|---|---|---|---|---|
| | cys-missense | non- cys-missense | p-value | cys-missense | non cys-missense | p-value |
| SV dilatation | 23/34 (67.6%) | 9/24 (37.5%) | **0.0327** | 6.3 ± 5.6 | 8.6 ± 6.3 | ns |
| MVP | 18/34 (52.9%) | 11/24 (45.8%) | ns | 6.5 ± 5.6 | 10.5 ± 5.8 | ns |
| TVP | 25/34 (73.5%) | 10/24 (41.7%) | **0.0281** | 6.3 ± 4.9 | 8.2 ± 7.6 | ns |
| PA dilatation | 2/34 (5.9%) | 4/23 (17.4%) | ns | 2.1 ± 1.7 | 5.4 ± 6.2 | ns |
| David | 4/34 (11.8%) | 1/24 (4.2%) | ns | 13.6 ± 6.2 | 10.9 ± 0.0 | ns |
| Medication | 24/34 (70.6%) | 7/24 (29.2%) | **0.003** | 7.5 ± 5.3 | 7.7 ± 5.2 | ns |
| Dural ectasia | 13/34 (38.2%) | 7/24 (29.2%) | ns | 10.5 ± 5.7 | 11.8 ± 3.7 | ns |
| High arched palate | 18/34 (52.9%) | 13/24 (54.2%) | ns | 7.9 ± 5.0 | 10.6 ± 4.9 | ns |
| Facial feature | 13/34 (38.2%) | 9/24 (37.5%) | ns | 5.1 ± 5.6 | 7.6 ± 6.6 | ns |
| Arm-span-to-height ratio > 1.05 | 5/34 (14.7%) | 9/24 (37.5%) | ns | 10.1 ± 5.3 | 11.9 ± 4.6 | ns |
| Pectus excavatum | 3/34 (8.8%) | 3/24 (12.5%) | ns | 6.9 ± 6.2 | 8.4 ± 10.0 | ns |
| Pectus carinatum | 9/34 (26.5%) | 5/24 (20.8%) | ns | 8.4 ± 4.0 | 12.7 ± 4.8 | ns |
| scoliosis | 11/34 (32.4%) | 10/24 (41.7%) | ns | 8.4 ± 4.9 | 11.3 ± 4.2 | ns |
| Wrist and thumb sign | 3/34 (8.8%) | 3/24 (12.5%) | ns | 8.5 ± 3.8 | 11.7 ± 5.4 | ns |
| Foot deformity | 22/34 (64.7%) | 11/24 (45.8%) | ns | 6.6 ± 5.0 | 9.8 ± 5.8 | ns |
| Reduced elbow-extension | 5/34 (14.7%) | 4/24 (16.7%) | ns | 12.9 ± 7.1 | 6.5 ± 5.0 | ns |
| Ectopia lentis | 15/34 (44.1%) | 6/24 (25.0%) | ns | 5.8 ± 5.3 | 9.8 ± 5.7 | ns |
| Myopia | 7/34 (20.6%) | 4/23 (17.4%) | ns | 3.7± 2.2 | 9.6 ± 4.8 | **0.0245** |
| Skin striae | 5/34 (14.7%) | 8/24 (33.3%) | ns | 13.2 ± 5.2 | 13.3 ± 2.1 | ns |
| Hernia | 2/34 (5.9%) | 1/24 (4.2%) | ns | 5.7 ± 1.6 | 4.4 ± 0 | ns |
| pneumothorax | 2/34 (5.9%) | 1/24 (4.2%) | ns | 15.6 ± 0.0 | 14.8 ± 0 | **0.025** |

| | | | | | | |
|---|---|---|---|---|---|---|
| Systemic score ≥ 7 | 10/32 (31.3%) | 9/23 (39.1%) | ns | 10.6 ± 5.9 | 9.7 ± 4.8 | ns |

**Table S5** Cardiovascular genotype-phenotype correlations (YOP, year of publication; cys-missense, missense mutations involving a cysteine; non-cys, missense mutations not involving a cysteine; PTC, premature termination codon; HI, hapoinsufficiency; DN, dominant-negative; cardiovasc, cardiovascular; SV-Dil, sinus of Valsalva dilatation; PA-Dil, pulmonary artery dilatation; TVP, tricuspid valve prolapse; MVP, mitral valve prolapse).

| Authors | YOP | Results |
|---|---|---|
| Loeys et al. | 2004 | No difference of cardiovasc. involvement (cys-miss/PTC) |
| Waldmüller et al. | 2007 | No difference of cardiovasc. involvement (cys-miss/PTC) |
| Detaint et al. | 2010 | More aortic dilatation, aortic events, and MVP in cys-missense |
| Baudhuin et al. | 2014 | More+earlier aortic events in in truncating+splicing variants than missense |
| Aubart et al. | 2015 | More aortic dilatation if low level of Wild-type *FBN1* mRNA |
| Yang et al. | 2016 | More aortic involvement in truncating or splicing variants than missense |
| Franken et al. | 2017 | More aortic phenotype in HI (larger, more rapid dilatation, increased risk of death and dissections) than in DN |
| Salvi et al. [53] | 2017 | Same aortic stiffness in HI and DN |
| Seo et al. | 2018 | More aortic dilatation in cys-missense than in non-cys-missense |
| Becerra-Munoz et al. | 2018 | More aortic events in truncating (nonsense, frameshift) than in missense |
| Takeda et al. | 2018 | More SV-Dil, aortic events in HI than DN<br>More SV-Dil, aortic events in DN-cys than DN-non-cys<br>Same risk aortic events in HI and DN-cys |
| Li et al. | 2019 | More aortic dissection in HI than DN |
| Gao et al. | 2019 | More cardiovasc. involvement in PTC than in non-PTC<br>More cardiovasc. involvement in cys-missense<br>More cardiovasc. involvement in exons 43–65 |
| Xu et al. | 2020 | More aortic dissection in HI (frameshift + nonsense) than in missense |
| Stark et al. | 2020 | More SV-Dil, TVP, medication in cys-missense than in non-cys-missense<br>Earlier PA-Dil in missense/in-frame than in nonsense/frameshift |

**Table S6** Ocular genotype-phenotype correlations (YOP, year of publication; EL, ectopia lentis, cys-missense, missense mutations involving a cysteine; non-cys, missense mutations not involving a cysteine; PTC, premature termination codon; HI, hapoinsufficiency; DN, dominant-negative)

| Authors | YOP | Results |
|---|---|---|
| Schrivjer et al. | 1999<br>2002 | More EL in cys-missense than in PTC |
| Comeglio et al. | 2002 | More EL in missense exons 1–15 |

| | | |
|---|---|---|
| Biggin et al. [54] | 2004 | More EL in cys-missense than in PTC |
| Loeys et al. | 2004<br>2007 | More EL in cys-missense than in PTC |
| Comeglio et al | 2007 | More EL in cys-missense than nonsense/frameshift |
| Rommel et al. | 2005 | More EL in cys-missense than PTC or non-cys missense |
| Faivre et al. | 2007 | More EL in cys-missense than in other missense |
| Aubart et al. | 2015 | More EL if low level of wild-type *FBN1* mRNA |
| Baudhuin et al. | 2015 | More EL in cys-missense than in other missense |
| Seo et al. | 2018 | More EL in missense than in other mutations |
| Becerra-Munoz et al. | 2018 | More EL in missense than nonsense, frameshift |
| Li et al. | 2019 | More EL in DN than in HI<br>More EL in cys-missense than in PTC |
| Gao et al | 2019 | More EL in cys-missense<br>More EL in non-PTC than PTC<br>More EL in missense than frameshift |
| Stark et al. | 2020 | More EL in missense than in nonsense/frameshift<br>Earlier myopia in cys-missense than in non-cys-missense |

**Table S7** Skeleton, skin, lung and dural genotype-phenotype correlations (YOP, year of publication; cys-missense, missense mutations involving a cysteine; non-cys, missense mutations not involving a cysteine; PTC, premature termination codon; HI, hapoinsufficiency; DN, dominant-negative)

| Authors | YOP | Results |
|---|---|---|
| Schrivjer et al. | 2002 | More skeletal involvement in PTC than in cys-missense |
| Loeys et al. | 2004 | More skeletal in PTC than in cys-missense |
| Rommel et al | 2005 | More skeletal in splice than PTC |
| Faivre et al. | 2007 | More skeletal involvement in PTC than in in-frame<br>More skin involvement in PTC than in in-frame |
| Comeglio et al. | 2007 | More skeletal involvement in nonsense/frameshift than cys-missense |
| Haine et al. | 2015 | More musculoskeletal in PTC than in in-frame |
| Aubart et al. | 2015 | More pectus abnormality if low level of wild-type *FBN1* mRNA |
| Franken et al. | 2015 | More pectus carinatum in HI than DN<br>More dural ectasia in HI than DN<br>More skin striae in HI than DN |
| Seo et al. | 2018 | Less dural ectasia in missense mutations than other variants |
| Becerra-Munoz et al. | 2018 | More positive systemic score in truncating (nonsense, frameshift) than in missense |
| Stark et al. | 2020 | More pectus excavatum in splicing than missense/in-frame<br>Earier hernias in missense/ in-frame than splicing<br>More dural ectasia in nonsense/frameshift than missense |
