# Supplementary material for: Genotype–Phenotype Correlation in Children: The Impact of FBN1 Variants on Pediatric Marfan Care
Source: Genes (Basel). 2020 Jul 15;11(7):799. doi: 10.3390/genes11070799 (PMC7397236; doi:10.3390/genes11070799)
Supplement: Supplementary file 1 [file genes-11-00799-s001.zip › supplement Data-variants.pdf]

<u>Supplementary Data</u>
FBN1 variants of our pediatric Marfan collective included in this study.

**(1) variants affecting a single or several codons, such as missense variants and in-frame deletions, that leave the reading frame intact. Missense variants have been subdivided into those destroying or creating a cysteine and those affecting another highly conserved amino acid residue**	
**cys-missense**	
c.184C>T	p.(Arg62Cys)
c.304T>C	p.(Cys102Arg)
c.496T>C	p.(Cys166Arg)
c.718C>T	p.(Arg240Cys)
c.718C>T	p.(Arg240Cys)
c.1663T>C	p.(Cys555Arg)
c.2342G>A	p.(Cys781Tyr)
c.2688T>G	p.(Cys896Trp)
c.2722T>C	p.(Cys908Arg)
c.2849G>T	p.(Cys950Phe)
c.2860C>T	p.(Arg954Cys)
c.3419G>A	p.(Cys1140Tyr)
c.3533A>G	p.(Tyr1178Cys)
c.4015T>C	p.(Cys1339Arg)
c.4172G>T	p.(Cys1391Phe)
c.4506T>G	p.(Cys1502Trp)
c.4538G>C	p.(Cys1513Ser)
c.4538G>C	p.(Cys1513Ser)
c.5009A>G	p.(Tyr1670Cys)
c.5009A>G	p.(Tyr1670Cys)
c.5020T>A	p.(Cys1674Ser)
c.5497T>G	p.(Cys1833Gly)
c.6049T>C	p.(Cys2017Arg)
c.6490T>A	p.(Cys2164Ser)
c.6628T>C	p.(Cys2210Arg)
c.7094G>A	p.(Cys2365Tyr)
c.7094G>A	p.(Cys2365Tyr)
c.7094G>A	p.(Cys2365Tyr)
c.7094G>A	p.(Cys2365Tyr)
c.7252T>C	p.(Cys2418Arg)
c.7252T>C	p.(Cys2418Arg)
c.7648T>G	p.(Cys2550Gly)
c.7814G>A	p.(Cys2605Tyr)
c.7976G>A	p.(Cys2659Tyr)

**non-cys-missense**	
c.248C>T	p.(Pro83Leu)
c.467A>G	p.(Asn156Ser)
c.640G>A	p.(Gly214Ser)
c.821C>A	p.(Pro274His)
c.3037G>A	p.(Gly1013Arg)
c.3463G>A	p.(Asp1155Asn)
c.4340T>G	p.(Ile1447Ser)
c.5339G>C	p.(Gly1780Ala)
c.5431G>A	p.(Glu1811Lys)
c.6055G>A	p.(Glu2019Lys)
c.6055G>A	p.(Glu2019Lys)
c.6388G>A	p.(Glu2130Lys)
c.6388G>A	p.(Glu2130Lys)
c.6431A>G	p.(Asn2144Ser)
c.6617A>T	p.(Asp2206Val)
c.6700G>A	p.(Val2234Met)
c.7606G>A	p.(Gly2536Arg)
c.7606G>A	p.(Gly2536Arg)
c.7661G>A	p.(Arg2554Gln)
c.7661G>A	p.(Arg2554Gln)
c.7661G>A	p.(Arg2554Gln)
c.7754T>C	p.(Ile2585Thr)
c.7858G>A	p.(Ala2620Thr)
c.8055C>A	p.(His2685Gln)
**In-frame**	
c.699_701delTGG	p.(Gly234del)
c.5076_5078delAAG	p.(Arg1692del)
c.5076_5078delAAG	p.(Arg1692del)
c.6038_6163del	p.(Ile2014_Asp2055del)
**(2) splice site variants, including intronic variants affecting one of the two invariable nucleotides of the splice acceptor or donor site, other intronic variants for which the in silico tools varseak/HumanSplicingFinder/MaxEntScan predicted an effect on pre-mRNA splicing, and a synonymous variant for which aberrant pre-mRNA splicing has been experimentally demonstrated.**	
**splicing**	
c.247+1G>A	
c.247+1G>A	
c.1589-2A>C	
c.2168-1G>C	
c.2168-1G>T	

c.3083-2A>C	
c.3338-2A>G	
c.3338-3_3346delCAGATATTGATG	
c.4211-1G>A	
c.4817-1G>T	
c.4460-8G>A	
c.4943-1G>A	
c.4943-1G>A	
c.6453C>T	p.Cys2151=
**(3) variants introducing a premature stop codon, such as nonsense and frameshift variants, and one deletion of the entire FBN1 gene, all likely leading to loss-of-function**	
**frameshift**	
c.532delG	p.(Glu178Lysfs*12)
c.653dupG	p.(His219Profs*4)
c.1383_1390dup	p.(Arg464Glnfs*118)
c.3220_3223delCAAT	p.(Cys1074Alafs*13)
c.3929delG	p.(Gly1310Alafs*103)
c.4639dupA	p.(Thr1547Asnfs*6)
c.4726_4728delinsGTACTCCT	p.(Met1576Valfs*7)
c.4726_4728delinsGTACTCCT	p.(Met1576Valfs*7)
c.4726_4728delinsGTACTCCT	p.(Met1576Valfs*7)
c.5259_5262delAGGC	p.(Gly1754Leufs*138)
c.5722delA	p.(Thr1908Glnfs*22)
c.5817delG	p.(Asn1940Ilefs*40)
c.7039_7040delAT	p.(Met2347Valfs*19)
c.7039_7040delAT	p.(Met2347Valfs*19)
c.7148delC	p.(Thr2383Metfs*15)
c.7148delC	p.(Thr2383Metfs*15)
c.7945_7946delAT	p.(Ile2649Glnfs*2)
c.8524_8528delCTTAA	p.(Leu2842Profs*7)
**nonsense**	
c.1090C>T	p.(Arg364*)
c.1546C>T	p.(Arg516*)
c.2670C>A	p.(Cys890*)

c.2670C>A	p.(Cys890*)
c.3797dup	p.(Tyr1266*)
c.6169C>T	p.(Arg2057*)
c.7045C>T	p.(Gln2349*)
c.7180C>T	p.(Arg2394*)
c.7240C>T	p.(Arg2414*)
c.8386G>T	p.(Glu2796*)
**deletion of the entire *FBN1* gene**	
15.q21.1q21.2(48,293,311_49,694,462)x1	(hg19, GRCh37)
